# From promise to pitfalls: immunological lessons from dengue vaccines and their implications

**DOI:** 10.1038/s41541-026-01400-4

**Published:** 2026-02-14

**Authors:** Cassia F. Estofolete, Marielena V. Saivish, Maurício L. Nogueira, Nikos Vasilakis

**Affiliations:** 1 Laboratório de Pesquisas em Virologia, Departamento de Doenças Dermatológicas, Infecciosas e Parasitárias, Faculdade de Medicina de São José do Rio Preto, São José do Rio Preto, SP Brazil; 2 Laboratório Nacional de Biociências, Centro Nacional de Pesquisa em Energia e Materiais (CNPEM), Campinas, SP Brazil; 3 Department of Pathology, University of Texas Medical Branch, Galveston, TX USA; 4 Center for Vector-Borne and Zoonotic Diseases, University of Texas Medical Branch, Galveston, TX USA; 5 Institute for Human Infection and Immunity, University of Texas Medical Branch, Galveston, TX USA

**Keywords:** Diseases, Immunology, Microbiology

## Abstract

Dengue continues to pose a significant global health threat. However, the development of a safe and effective tetravalent vaccine is impeded by complex serotype-specific and cross-reactive immune responses. This review assesses licensed and advanced dengue vaccines, emphasizing serostatus-dependent safety, serotype imbalance, and the durability of protection. It also critically evaluates immunological correlates of protection and addresses remaining obstacles, such as vector-mediated immune modulation and orthoflavivirus cross-reactivity, to guide future dengue vaccine strategies.

## Introduction

Dengue virus (DENV), recently reclassified as *Orthoflavivirus dengue* within the *Flaviviridae* family^1^, remains one of the most pervasive and intractable arthropod-borne viral threats to human health. The global burden is staggering: an estimated 390 million infections occur annually, of which roughly 96 million manifest clinically, spanning from self-limiting febrile illness to severe dengue characterized by plasma leakage, hemorrhage, and multi-organ dysfunction^2,3^. The virus comprises four antigenically distinct serotypes (DENV-1 to -4), each capable of causing severe disease, thereby necessitating immunity to all serotypes for effective long-term protection.

The principal vectors, *Aedes aegypti* and *Ae. albopictus*, have expanded their range dramatically over recent decades, propelled by rapid unplanned urbanization, intensified global trade, increased human mobility, and shifting environmental conditions^4^. This ecological expansion has transformed dengue from a predominantly tropical disease into one with established autochthonous transmission in subtropical and even temperate regions, including parts of Europe and the continental United States^5,6^. Consequently, hyperendemic transmission is now entrenched in large swaths of the tropics, where sequential infections by different serotypes are common and the majority of the global population remains at risk. Epidemiological modeling projects that over 5 million symptomatic cases and at least 5000 deaths occur annually in more than 80 countries^3^, with a concomitant rise in disability-adjusted life years (DALYs) and substantial economic costs^7,8^.

Clinically, dengue presents a broad phenotypic spectrum. Mild disease manifests with fever, myalgia, arthralgia, retro-orbital pain, headache, and rash, whereas severe dengue can rapidly progress to shock, hemorrhage, and neurological or cardiac complications^9,10^. Mortality may exceed 20% without timely intervention but can be reduced below 1% with optimal supportive care^10^. No antiviral therapy is currently available; public health control relies primarily on vector management and, where available, vaccination^11^. The development of a safe, efficacious, and widely deployable dengue vaccine has proven scientifically challenging. Achieving durable protective immunity against each DENV serotype is fundamental for ensuring protection. A significant unresolved challenge is the operational definition of ‘balanced and durable protection’ and whether a single definition and threshold are applicable to DENV-1 through DENV-4, considering serotype-specific variations in immunodominance, transmission intensity, and durability. The absence of harmonized definitions hinders cross-trial comparisons and complicates the interpretation of serotype-specific protection over time. Incomplete or heterogeneous immunity across serotypes, resulting from waning antibody titers, antigenic differences, or immune imprinting, can lead to breakthrough infections. In certain contexts, this may increase disease severity through multiple immunological mechanisms, including (but not limited to) antibody-dependent enhancement (ADE)^12,13^, a phenomenon whereby cross-reactive, sub-neutralizing antibodies facilitate viral entry into Fc receptor–bearing cells, increasing viral replication and inflammatory damage^12,14^. Additionally, immunological phenomena, such as original antigenic sin and extensive orthoflavivirus cross-reactivity may bias B- and T-cell repertoires toward epitopes of the primary infecting serotype or heterologous orthoflaviviruses, potentially undermining the breadth of protection^15,16^.

Naturally acquired immunity to DENV integrates multiple layers of immune defense: type-specific and cross-reactive neutralizing antibodies targeting complex quaternary epitopes on the envelope protein; antibodies to conserved fusion-loop epitopes with varying functional consequences; and polyfunctional CD4+ and CD8 + T-cell responses that contribute to viral clearance and long-term immune memory^17,18^. An effective vaccine must recapitulate these protective elements while avoiding immunopathogenic pathways. This entails not only achieving high titers of broadly neutralizing antibodies but also generating balanced serotype-specific memory B cells and robust T-cell immunity that are maintained across genetically and immunologically diverse populations.

This review provides an expert, interpretive synthesis of dengue vaccine immunology. Evidence was purposively selected from high-impact clinical trials, long-term follow-ups, authoritative assessments, and mechanistic studies judged most decision-relevant to current vaccine design and policy. The selection process was non-systematic and reflects the authors’ expert appraisal. In cases where key studies diverged, randomized evidence and longer follow-up data are prioritized, and unresolved discrepancies are explicitly noted. To avoid ambiguity in terminology and measurements used throughout this review, Table 1 standardizes key definitions, clinical endpoints, and assay conventions relevant to dengue vaccine evaluation, including virologically confirmed dengue (VCD), hospitalized/severe disease, baseline serostatus, neutralization breadth, avidity, memory B-cell breadth, CD8⁺ T-cell polyfunctionality, and post-vaccination viremia. Harmonizing these definitions a priori, allows for consistent interpretation of subsequent comparisons (Tables 2–4) and the proposed correlates framework can be interpreted consistently across trials, serotypes and laboratories, thus minimizing misclassification due to heterogeneous protocols and readouts. Throughout this review, the term ‘viremia’ is used as reported in the original studies and may refer either to (i) RNAemia measured by RT-PCR (viral genomes) or (ii) infectious viremia measured by cell culture based assays (e.g., plaque or focus forming assay). Because these assays capture different biological outputs, the assay type is specified when interpreting results, and direct cross-study comparisons are avoided when methods differ.

**Table 1:** Standardized key definitions, clinical endpoints, and assay conventions relevant to dengue vaccine evaluation

Term / Metric	Working definition (as used here)	Why it matters for vaccine design	Recommended measurement / assay	Common pitfalls / notes
**Virologically confirmed dengue (VCD)**	Symptomatic dengue meeting trial case definition with RT-PCR and/or NS1 positivity during the acute window	Primary efficacy endpoint in pivotal trials; anchors VE estimates	RT-PCR on acute sera; NS1 antigen tests per protocol window	Heterogeneous sampling windows reduce sensitivity; misclassification if only serology is used
**Hospitalized VCD**	VCD requiring hospital admission per trial adjudication	Proxy for clinically meaningful protection and health system burden	Same as VCD + verified admission criteria	Admission thresholds vary by site; requires blinded adjudication
**Severe dengue**	VCD meeting WHO severity criteria (shock, severe bleeding, organ impairment)1	Captures prevention of the worst outcomes	WHO 2009 adjudication with clinical labs	Criteria application can vary; ensure prespecified charter
**Baseline serostatus**	Seropositive vs seronegative at enrollment (neutralization or IgG cutoff)	Modifies VE and safety signals; guides policy and indication	PRNT or validated IgG assay at baseline	Assay cutoffs differ; misclassification affects subgroup of vaccinal efficacy (VE)
**PRNT**_**50**_ **vs PRNT**_**90**_	Serum dilution reducing plaques by 50% vs 90%	PRNT_90_ is more specific; PRNT_50_ refers to the baseline	Standardized PRNT across four serotypes	Inter-lab variability; not an epitope-resolved readout
**Neutralization breadth index**	Composite capturing potency across DENV-1-4, optionally weighted by epitope quality	Predicts protection better than a single mean titer	PRNT_50_/PRNT_90_ per serotype, summarized as breadth score	Can mask serotype gaps if averaged; report per serotype + index
**Envelope dimer epitope (EDE)-like epitope targeting**	Antibody specificity to quaternary E-dimer epitopes	Tracks with “quality” and sterilizing activity; mitigates ADE risk	E-dimer competition ELIZA; epitope-resolved neutralization	Not captured by standard PRNT; needs specialized reagents
**Avidity index**	Strength of antigen–antibody binding (chaotrope-resistant fraction)	Surrogate of GC maturation; associates with durability	Avidity ELIZA (e.g., urea wash) vs E antigens	Assay conditions must be standardized; not a direct correlation of protection (CoP)
**Memory B-cell (MBC) breadth**	Proportion of MBCs recognizing DENV-1-4 antigens	Predicts recall and long-term protection	B-cell ELISSPOT or antigen-labeled flow cytometry	Antigen panel must be balanced; timepoint matters (6–12 mo)
**CD8⁺ T-cell polyfunctionality (NS-focused)**	% CD8⁺ producing ≥2 cytokines to NS3/NS5 pools	Complements antibodies; may reduce breakthrough at equal PRNT	Intracellular cytokine Staining (ICS)/flow cytometry or ELISpot with NS peptide pools	Standardization of peptide pools and gating is critical
**Post-vaccination viremia / replication**	Proportion with detectable vaccine-virus viremia at set days post-dose	Correlates with early immunogenicity for live-attenuated vaccines (LAV)	qRT-PCR and/or infectious virus assays (days 7/12/20)	Report exact timepoints and thresholds; define +/++/+++ scale
**Serotype-resolved Vaccine efficacy (VE)**	VE for DENV-1,-2,-3,-4 separately with 95% CIs	Detects imbalance hidden by overall VE	Trial analysis by RT-PCR-typed episodes	Wide confidence intervals (CIs) when circulation is low; mark NA when not estimable
**Durability (year-by-year VE)**	VE trajectory by year post-vaccination	Guides booster/prime–boost strategies	Annual VE estimates up to 4–5 years	Serotype mix changes over time; interpret with circulation data
**Breakthrough virology & antigenic cartography**	Genotype/serotype mapping of breakthroughs vs vaccine antigenic space	Distinguishes waning vs antigenic mismatch	Sequencing + antigenic maps	Requires centralized labs; needs harmonized panels

Note. We use WHO 2009 severity criteria; plaque reduction neutralization test (PRNT) readouts should be reported per serotype and according to assay stringency; in settings where the incidence of a given serotype is too low to allow reliable estimation, VE is marked NA and interpreted with caution.

^1^ For TAK-003 trials, severe dengue disease endpoints were assessed using a dengue case adjudication committee (study-specific criteria) alongside protocol-defined clinical endpoints; definitions and adjudication procedures are described in the primary trial reports^35^.

**Table 2:** Description of immune and virological aspects of each vaccine

	DENV-1	DENV-2	DENV-3	DENV-4
**Vaccine**	**Serotype-specific neutralizing antibodies**^**#**^			
Dengvaxia® (DEN-YF17D)	-	-	-	Yes
Qdenga® (TAK-003)	-	Yes	-	
Butantan-DV (TV003/TV005)	Yes	Yes	Yes	Yes
	Vaccine virus replication after any dose of immunization*			
Dengvaxia® (DEN-YF17D)^*^	+	not detected	+	+++
Qdenga® (TAK-003) ^**^	+	+++	+	+
Butantan-DV (TV003/TV005) ^***^	+++	++++	+++	+++
	**Overall efficacy** (VE against symptomatic VCD)^##^			
Dengvaxia® (DEN-YF17D)	50.3%	42.3%	74%	77%
Qdenga® (TAK-003)	73.7%	97.7%	62.6%	inconclusive
Butantan-DV (TV003/TV005)	89.5%	69.6%	Not available	Not available

#Serotype-specific neutralizing antibodies are reported as described in the original studies and generally reflect the serotype(s) showing dominant neutralization at the specified post-vaccination timepoint (e.g., highest PRNT GMT and/or serotype-skew patterns). Where depletion/competition mapping was performed, this is noted; otherwise, values reflect PRNT-based dominance.

## Efficacy window/timepoint: CYD-TDV: pivotal Phase III active-phase primary analyses ( ~ 25 months); TAK-003: primary analysis window (12 months) and/or longer follow-up if explicitly stated; Butantan-DV: interim primary analysis ( ~ 2 years) (DENV-3/4 NA due to low circulation).

*Available up to 20 days after vaccination; **available up to 17 days after vaccination ***available 7 and 12 days after vaccination. GMT – Geometric mean titer.

Note: * The + representation (from + to ++++) is a qualitative method of quantification to describe the percent of individuals detected with virus replication post vaccination: 1–25%: +; 26–50%: ++; 51–75%: +++; 76–100%: ++++.

**Table 3:** Description of immune and virological aspects of each vaccine

Vaccine (platform)	Antigenic content / NS proteins	Doses & schedule	Regions & follow-up	Primary endpoint	Overall VE [95% CI] (window)	VE by serotype [95% CI]	Baseline serostatus effect	Hospitalization / Severe VE	Durability (headline)	Safety notes	Neutralization assay
**CYD-TDV (Dengvaxia®; chimeric YF17D)**	prM/E of DENV-1-4 on YF17D backbone; no dengue NS	3 doses (0–6–12 mo)	Asia (CYD14), Latin America (CYD15); 25-mo active; LTFU 6 y+	Laboratory confirmed dengue (VCD; RT-PCR/NS1)	~65.6% (60.7–69.9), pooled active-phase	D1: 58.4% (47.7–66.9) D2: 47.1% (31.3–59.2) D3: 73.6% (64.4–80.4) D4: 83.2% (76.2–88.2)	Marked dependence: higher VE in seropositives; safety concern in seronegatives: seropositive: 81.9% (67.2–90.0) vs seronegative: 52.5% (5.9–76.1), pooled 9–16 y during active surveillance. Increased risk of severe dengue/hospitalization in vaccinated seronegatives reported in long-term follow-up.	Hosp: 80.8% (70.1–87.7); Severe: 93.2% (77.3–98.0)	Waning over time; seroneg. hazard signal in years 3–5 triggered test-before-vaccination policy	Seronegative recipients had increased risk of hospitalized/severe dengue vs placebo	PRNT_50/90_ (varied by lab)^24–26,31^
**TAK-003 (Qdenga®; DENV-2 PDK-53–derived LAV)**	Structural for all 4; NS from DENV-2 in the backbone	2 doses (0, 3 mo)	Multiregional (Eight endemic countries); 12-mo primary; 4.5-y follow-up	VCD; key secondary = hospitalized/severe VCD	12 mo: 80.2% (73.3–85.3)	12 mo: D1: 73.7% D2: 97.7% D3: 62.6% (D4: inconclusive at 12 mo)	Benefit in both seropositive and seronegative; magnitude higher in seropositives: VCD VE at 18 mo: seronegative 66.2% (49.1–77.5) vs seropositive 76.1% (68.5–81.9); cumulative at ~4.5 y: 53.5% (41.6–62.9) vs 64.2% (58.4–69.2).	4.5 y: Hosp VE 84.1% (77.8–88.6) overall	4.5 y: Overall VE 61.2% (56.0–65.8); protection strongest vs DENV-1/2; weaker vs DENV-3/4 esp. in seronegatives	No new major safety signals in Phase III; routine reactogenicity	PRNT_50_ (trial network); epitope quality not routinely captured ‘^35–37^
**Butantan-DV (TV003/TV005 mix; NIH-LAV)**	Attenuated DENV-1,-3,-4; chimeric DENV-2/4; includes dengue NS per component	Single dose (Brazil Phase III)	Brazil; ~2-year interim; DENV-1/2 dominated circulation	VCD; secondary = hospitalized/severe VCD	79.6% (70.0–86.3), ~2 y	D1: 89.5% (78.7–95.0) D2: 69.6% (50.8–81.5) D3/D4: NA (not circulating)	Higher VE in seropositives (age-stratified estimates reported): at 2 y: dengue-naïve 73.6% (57.6–83.7) vs previously exposed 89.2% (77.6–95.6).	Age 2–6 y: 80.1% (66.0–88.4); 7–17 y: 77.8% (55.6–89.6); 18–59 y: 90.0% (68.2–97.5)	Ongoing follow-up to 5 y (durability & breakthrough genotyping/antigenic cartography)	No major safety signal to date	PRNT_50_ (trial network)^46^

Notes: CI = 95% confidence interval, *VE* vaccine efficacy, *NA* not estimable due to lack of circulation. Serotype-specific VE for CYD-TDV shown from pooled Phase III active-phase analyses; Dengvaxia is restricted to seropositive populations due to seronegative risk signal. TAK-003 shows robust early protection and sustained prevention of hospitalization to 4.5 y, with heterogeneity by serotype and baseline serostatus over time. Butantan-DV shows strong efficacy vs DENV-1/2 in Brazil with DENV-3/4 unmeasured so far; extended follow-up is underway.

**Table 4:** Minimal composite correlate (proposal^#^) and practical assays for dengue vaccine trials

Component	What it captures	Operational metric	Assay/method	Sampling window	Example decision rule
**Neutralization breadth index (Tier A)**	Potency and coverage across DENV-1-4, adjusted for epitope quality	Breadth-weighted PRNT_50/90_ across 4 serotypes **×** EDE-competition factor	PRNT_50/90_; E-dimer competition ELIZA; epitope-resolved neutralization panel	Peak (day 28–60) + durability (6, 12, 24 mo)	Index ≥ prespecified X; failure if below X and narrowness to 1–2 serotypes
**Avidity (Tier A add-on)**	GC maturation / Ab avidity	Chaotrope-based avidity index vs E antigens	Avidity ELIZA (e.g., urea wash)	Day 60; 6–12 mo	Avidity ≥ Y linked to lower breakthrough rates
**Memory B-cell breadth (Tier B)**	Recall potential across serotypes	% antigen-specific MBC recognizing E/NS1 for DENV-1-4	B-cell ELISpot or Ag-labeled flow cytometry	6–12 mo	Breadth index ≥ Z predicts year-2+ protection
**CD8⁺ T-cell polyfunctionality (Tier B)**	NS-directed cellular breadth	% polyfunctional CD8⁺ (IFNγ/IL-2/TNF-α) to NS3/NS5 peptide pools	ICS/flow cytometry; ELISpot	Day 28–90	Composite CD8⁺ score ≥ W associated with lower VCD odds at matched PRNT

#Conceptual framework (hypothesis-generating; not clinically validated).

## Immunological Challenges in Dengue Vaccine Development

**Fig. 1:**
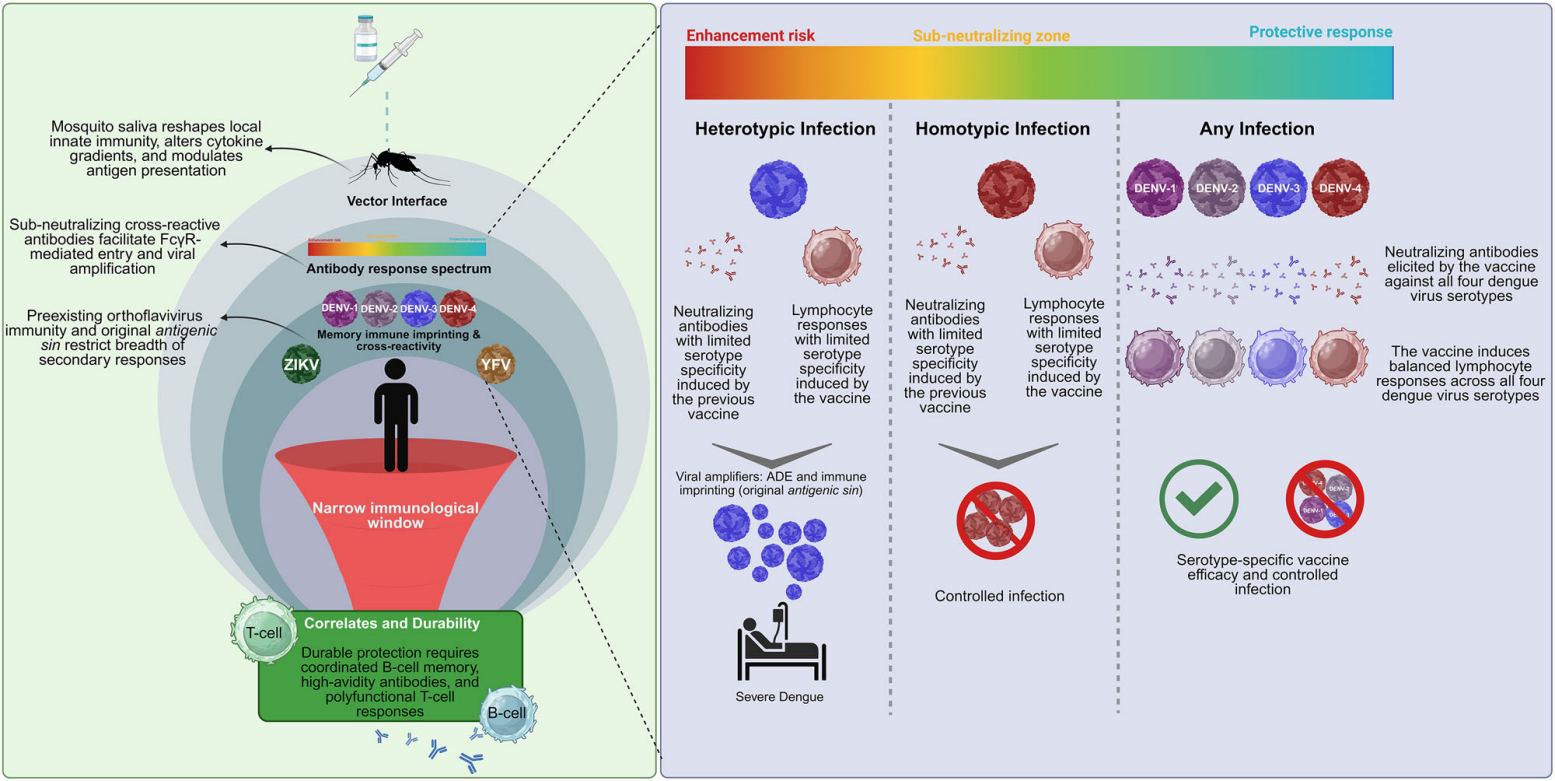
Bottlenecks in dengue vaccinology: the narrow window between protection and enhancement. This schematic integrates the multilevel barriers that shape dengue vaccine performance. The left panel depicts the cascade from mosquito-driven innate modulation to ADE and memory bias. The funnel highlights the narrow immunological window where protection and enhancement overlap. The right panel illustrates outcomes across infection contexts. In heterotypic scenarios, sub-neutralizing or cross-reactive antibodies may amplify viral replication via Fcγ mediated pathways consistent with ADE and antigenic imprinting, increasing severe dengue risk. Homotypic immunity yields serotype-limited but protective control, whereas balanced tetravalent immunity confers broad protection without enhancement. Durable vaccine efficacy depends on coordinated B-cell memory, high-avidity antibodies, and polyfunctional T-cell responses. Created in BioRender. Vogel Saivish, M. (2026) https://BioRender.com/r4sphjh.

Dengue vaccine development has faced persistent obstacles due to the unique immunological complexities associated with DENV infection. A primary and central challenge is achieving balanced and long-lasting protection against all four serotypes. In natural infection, sequential exposure to heterologous serotypes is a major risk factor for severe clinical disease, which appears to be multifactorial process influenced by viral characteristics, host genetics, and immunological background. In this context, ADE has been identified as a potential contributor in certain contexts, along with T-cell-mediated immunopathology, complement activation, cytokine-driven vascular permeability, and other immune regulatory mechanisms that can promote infection of myeloid cells and amplify inflammatory responses^12–14^. Although it may not be feasible to completely eliminate antibodies with enhancing potential, a practical goal in vaccinology is to direct immune responses toward high-avidity and quaternary-epitope neutralization, favorable Fc effector functions, and durable immune memory that sustains protective immunity above risk thresholds as antibody titers decline (Fig. 1)^12,19^.

Another major hurdle lies in the phenomena of original antigenic sin and cross-reactivity with other orthoflaviviruses. Primary exposure to one DENV serotype can bias subsequent B- and T-cell responses toward epitopes of the original infecting strain, limiting the breadth of immunity when a different serotype is encountered^14^. Similarly, prior immunity to related orthoflaviviruses, including Zika and yellow fever, may redirect responses toward cross-reactive epitopes, complicating vaccine performance in orthoflavivirus-endemic regions^20^ (Fig. 1).

Equally important is the identification of reliable correlates of protection. While neutralizing antibody titers are commonly used as a benchmark, accumulating evidence demonstrates that serotype-specific neutralization does not always correlate with clinical protection^21^. Instead, immunity likely involves a combination of factors, including the quality and durability of antibody responses, memory B-cell repertoires, and the magnitude, functionality, and breadth of CD4+ and CD8 + T-cell responses^18^. Defining these correlates is critical to guiding both vaccine design and evaluation in clinical trials. Finally, the context of natural transmission by mosquito bite adds another layer of complexity. Components of mosquito saliva can modulate host immune responses at the inoculation site, altering viral kinetics and shaping adaptive immunity^22^. Most preclinical and clinical studies rely on needle inoculation, which may not accurately capture these early immunological dynamics. This gap underscores the importance of considering vector–host–virus interactions when evaluating vaccine efficacy. Together, these factors define the central immunological barriers to dengue vaccine development. A successful vaccine must simultaneously overcome serotype diversity, preexisting immunity, multifactorial immunopathogenic risks following heterologous infection (including, but not limited to, ADE), and vector-mediated modulation of infection, requirements that few other viral vaccines face in such combination. These multilevel barriers converge conceptually within what has been termed the “narrow immunological window” of dengue vaccinology, a space where protective and enhancing immune responses coexist in delicate balance (Box 1).

BOX 1. The Immunological Narrow Window: Lessons from Dengue Vaccinology for Complex Viral Pathogens *The concept of the “narrow immunological window” encapsulates one of the defining paradoxes of dengue vaccinology: the same antibodies that protect against infection can, under specific stoichiometric and kinetic conditions, enhance viral entry and immunopathology*.

In dengue, protection and enhancement are separated by a remarkably fine quantitative and qualitative boundary. High-avidity, quaternary-epitope antibodies neutralize infection by blocking virion fusion or destabilizing the E-dimer interface. Yet, when antibody concentrations fall into a sub-neutralizing range (or when cross-reactive memory B cells dominate recall responses) these antibodies can facilitate FcγR-mediated uptake into monocytes and macrophages, amplifying replication and inflammatory signaling. This duality defines the “narrow immunological window,” a space where vaccine-induced immunity can shift from protective to pathogenic. The phenomenon is not merely quantitative but structural and contextual. Antibody avidity, epitope topology, and Fc subclass determine whether Fc engagement results in viral clearance or enhancement. Vector-driven innate modulation, original antigenic sin, and orthoflavivirus cross-reactivity further narrow this window, especially in endemic populations with layered orthoflavivirus immunity. The dengue experience thus provides a cautionary blueprint for vaccine design against other complex pathogens, those where partial or imbalanced immunity can potentiate rather than prevent disease (e.g., Zika, RSV, SARS-CoV-2). Rational vaccinology must therefore aim not only for magnitude but for quality: durable, balanced, and epitope-precise immunity that remains on the protective side of this narrow window.

## Licensed Vaccines and Candidates: Immunological Profiles

The quest for a dengue vaccine has yielded three major candidates to date that reached licensure or advanced clinical evaluation: Dengvaxia® (CYD-TDV, Sanofi Pasteur), Qdenga® (TAK-003, Takeda), and the Butantan-DV (TV003/TV005, Butantan Institute/NIH). A NIH live-attenuated tetravalent vaccine platform (Merck’s version) has progressed to Phase 3 evaluation. However, due to the absence of detailed peer-reviewed efficacy/immunogenicity datasets in the public domain, this program is mentioned for completeness but is not included it in the comparative analyses. Each of these vaccines embodies both progress and pitfalls, underscoring the singular immunological challenges of dengue vaccinology.

### Dengvaxia®

Dengvaxia® (CYD-TDV, Sanofi Pasteur) was the first dengue vaccine to achieve regulatory approval, representing a milestone in arbovirus vaccinology but also one of its most sobering lessons. The vaccine is a live chimeric construct in which the prM and E structural genes of each dengue serotype are inserted into the attenuated yellow fever 17D backbone, thereby aiming to generate a tetravalent formulation capable of balanced immune stimulation^23^. This design leveraged the established safety record of YF17D while attempting to provide the breadth of protection necessary for dengue. However, clinical experience has demonstrated that this platform introduced profound immunological constraints that continue to shape the field. Large multicenter Phase III trials conducted across Asia and Latin America showed moderate overall efficacy (approximately 60% against symptomatic dengue) with considerable heterogeneity across serotypes and populations^24,25^. Protection was highest against DENV-4, intermediate against DENV-1 and DENV-3, and weakest against DENV-2^24^. These differences likely reflect both the intrinsic immunogenicity of the chimeric constructs and the interactions between vaccine-induced immunity and prior orthoflavivirus exposure in endemic populations^25^. Efficacy was markedly influenced by baseline serostatus: individuals with preexisting immunity derived significant protection, while seronegative recipients not only failed to achieve consistent benefit but also experienced increased risk of hospitalization and severe disease upon subsequent natural infection^26^. This paradox may be due to the induction of cross-reactive, sub-neutralizing antibody response that could, in certain contexts, increase the likelihood of enhancing antibody profiles during breakthrough infections. However, a definitive mechanism explaining the elevated risk observed in baseline seronegatives has not been established in vaccine recipients.

Immunological analyses have underscored several mechanistic explanations for these outcomes. First, Dengvaxia® induces an antibody response that is quantitatively high but qualitatively unbalanced, with DENV-4 immunodominance and weak induction of DENV-2-specific neutralizing antibodies^27^. Second, the chimeric design lacks dengue nonstructural proteins (NS proteins), limiting the breadth of CD8⁺ T cell responses that are critical for viral clearance and durable protective immunity^28^. This contrasts with natural infection, which elicits polyfunctional CD8⁺ T cells targeting NS3 and NS5 proteins, responses largely absent in CYD-TDV recipients^29^. Third, long-term follow-up has revealed waning neutralization titers over time, particularly in seronegative individuals, exacerbating the risk of ADE during subsequent exposure^30^. These features explain why protection was skewed toward individuals with prior dengue exposure, for whom the vaccine effectively acted as a booster, but detrimental in naive populations, where it may have primed for enhanced disease.

The public health consequences of these findings have been substantial. Following initial approval and rollout in the Philippines, post-licensure surveillance identified an excess risk of severe dengue in vaccinated children who were seronegative at baseline^26^. This resulted in suspension of vaccination programs and widespread public concern, underscoring the need for stringent risk stratification. In 2018, the World Health Organization revised its position, recommending Dengvaxia® only for individuals with confirmed prior infection, a requirement that limits its practicality in endemic regions where serostatus screening is logistically challenging. Despite these limitations, Dengvaxia® still remains licensed in some countries and provides valuable protection for previously infected individuals in whom the vaccine can reduce hospitalization and severe outcomes^31^. However, Sanofi has announced the discontinuation of manufacturing/distribution, and availability is expected to end after August 2026, subject to depletion of remaining stock according to country-specific timelines^32^ From a scientific standpoint, the Dengvaxia® experience highlighted the critical immunological principles that must guide all dengue vaccine development. It demonstrated that neutralizing antibody titers alone cannot serve as reliable correlates of protection; that balanced serotype coverage is indispensable; and that the absence of nonstructural antigens compromises cellular immunity and long-term durability^27–29^. More broadly, it revealed the ethical and epidemiological risks of large-scale deployment without full understanding of host–virus–vaccine interactions in diverse populations. Dengvaxia® therefore stands as both a breakthrough and a cautionary tale, shaping the trajectory of subsequent candidates by highlighting the perils of partial or unbalanced immunity in a disease where enhancement has been proposed as one potential contributor among multiple immunopathogenic pathways.

### Qdenga®

Qdenga® (TAK-003, Takeda Pharmaceuticals) is the second tetravalent dengue vaccine to reach licensure and has become a central case study in both the promise and the persistent challenges of dengue vaccinology. Unlike Dengvaxia®, which uses a yellow fever 17D backbone, Qdenga® is based on an attenuated DENV-2 strain (PDK-53) engineered to express the prM and E proteins of DENV-1, DENV-3, and DENV-4, thereby aiming to generate a live-attenuated chimeric tetravalent construct^33^. This strategy sought to circumvent the limitations of Dengvaxia® by retaining dengue nonstructural proteins (critical targets for CD8⁺ T-cell responses) while maintaining sufficient attenuation for safety^34^. Phase II and III clinical trials conducted in Asia and Latin America demonstrated encouraging efficacy against symptomatic VCD, particularly against DENV-2, with protection estimates nearing 97–98% during the 12-month primary analysis window. Efficacy was lower for DENV-1 ( ~ 74%) and DENV-3 ( ~ 63%), while results for DENV-4 were inconclusive due to limited case numbers^35^. Unlike Dengvaxia®, Qdenga® did not show increased risk of severe dengue in seronegative recipients, a difference that may be attributable to the inclusion of dengue nonstructural antigens and early evidence showing detectable replication from multiple components. However, subsequent analyses have suggested that post-vaccination viremia and the resulting type-specific neutralization can be dominated by the DENV-2 backbone component, consistent with immunodominance driven by differential replication^35,36^. Collectively, these findings suggest that Qdenga® may be safer and potentially more broadly deployable option for endemic regions.

Nevertheless, longer-term follow-up has revealed substantial caveats. The overall VE of Qdenga® against symptomatic VCD decreased from ~80% in the first year to ~62% at 3 years, while protection against hospitalization/severe outcomes remained the key clinically meaningful endpoint, and is summarized in Table 3^37^. The serotype imbalance also persisted: protection against DENV-2 remained robust, but efficacy against DENV-1 and DENV-3 declined, raising concerns regarding breakthrough infections in hyperendemic settings^36^. Immunological analyses suggest that DENV-2 replication dominates the post-vaccination viremia, overshadowing responses to the other serotypes, resulting in unbalanced neutralization profiles^21^. This shift compared to earlier reports may be attributable to differences in assay sensitivity/definitions of viremia, sampling intensity and timing, vaccine lots/formulations, and baseline immunity in enrolled populations. These factors can influence which components are detected and how replication leads to serotype-skewed immunogenicity^38,39^ Moreover, the quality of antibody responses has been questioned, as neutralization titers do not always correlate with clinical protection, echoing lessons from earlier candidates. Another unresolved issue is the durability and breadth of T-cell responses. While the inclusion of dengue NS proteins theoretically broadens CD8⁺ T-cell immunity, detailed immunoprofiling remains limited. Existing studies suggest that TAK-003 can induce broad and multifunctional dengue-specific T-cell responses (including NS-focused responses) and that these responses can persist beyond early post-vaccination timepoints. However, the specific contribution of these responses to clinical protection particularly in seronegative individuals remains incompletely defined^29,40–43^. In summary, efficacy has varied by serotype and baseline serostatus. Protection has been strongest and most durable against DENV-2, with more uncertainty for DENV-3/4 in baseline seronegatives individuals due to limited case numbers and wide confident intervals. These constraints should be clearly acknowledged when interpreting “overall” VE^35,36^.

From a regulatory and public health perspective, Qdenga® has been licensed in the European Union and several dengue-endemic countries, with rollout in regions where dengue represents a significant burden^44^, however, Qdenga^®^ is not currently licensed/approved in the United States^45^. Its approval without the restriction of baseline serostatus screening is a major advantage over Dengvaxia^®^, as it permits broader implementation in routine immunization programs. However, questions remain about its long-term effectiveness in real-world hyperendemic settings, where multiple serotypes co-circulate and herd immunity dynamics differ from controlled trial populations.

### Butantan-DV

Recently, the Butantan Institute, the NIH and Merck (MSD) reported the initial results of the Butantan-DV (TV003/TV005) vaccine, stemming from the phase III clinical trial conducted in Brazil with over 16,000 enrollees followed for 2 years^46^. This LAV was developed through the deletion of 30 and 31 contiguous nucleotides within the 3’UTR of wild-type DENV-1, -4^47–50^, and DENV-3^51^, respectively. As for the DENV-2 vaccine component, the prM and E proteins of DENV-4 were substituted with the corresponding genes of DENV-2^48–50,52^.

The vaccine candidate was subjected to extensive preclinical evaluation for viral replication and immunogenicity in rodents^53^, and non-human primates^47^, and their potential to be transmitted by *Aedes* species mosquitoes^54^. They all triggered an immune response in *Rhesus* macaques, confirming their immunogenicity and demonstrated lower replication rates than the wild-type virus, confirming successful attenuation^47,49–52^ and suggesting that transmission by mosquitoes may be unlikely^52,55^. Troyer et al. ^54^ experimentally demonstrated that mosquitoes feeding on vaccinated individuals with the monotypic DENV-4 vaccine candidate during the viremic phase were unable to transmit, as the virus was not able to disseminate from the midgut to the salivary glands. Collectively, these data supported further clinical development by showing immunogenicity and attenuation in preclinical models and limited potential for human transmission, providing the basis for further development.

### Phase I and II studies

Sequentially, the phase I and II studies assessed infectivity, viral replication kinetics and immunogenicity of each monovalent vaccine component. All monovalent formulations were well tolerated^49,56–58^, with rash and transient neutropenia being the most common symptoms, and fever reported in only one of the 241 participants. No participants exhibited dengue symptoms, despite the detection of levels of viremia in all participants, suggesting high viral attenuation. Seroconversion was ³ 80% for each serotype by day 42 post-vaccination^56,57,59^. The high tolerance and minimal adverse events reiterated a strong safety profile, whereas the high seroconversion rates supported immunogenicity across serotypes. However, immunogenicity measures alone should not be interpreted as evidence of clinical protection without endpoint-driven efficacy/effectiveness data or validated correlates of protection. The low levels of viremia suggest further studies are necessary to ensure consistent and robust immune responses across diverse populations.

The monovalent candidates were then evaluated together as tetravalent formulations, to determinate the most suitable candidates for safety, viral replication, and immunogenicity. Six monovalent components were combined into four tetravalent admixtures (TV001–TV004; 3.3 log10 PFU per component). TV001 and TV002 used the DENV-3 chimeric component rDEN3-3′D4Δ30, whereas TV003 and TV004 used rDEN3Δ30/31; TV001 and TV003 contained rDEN4Δ30, while TV002 and TV004 contained the more attenuated rDEN4Δ30-200,201. Across all four formulations, the DENV-1 and DENV-2 components were conserved (rDEN1Δ30 and rDEN2/4Δ30)^60^. All formulations were well tolerated and immunogenic, with a trivalent response in most individuals. TV003 induced high seroconversion rates in orthoflavivirus-naïve individuals. Lower seropositivity observed in African American participants was associated with lower post-vaccination viremia in that cohort, highlighting the importance of vaccine-virus replication for immunogenicity; however, these findings also underscore the need to better understand host and population factors when extrapolating immunogenicity across diverse groups^60,61^. The antibody magnitude alone does not establish balanced tetravalent immunity nor the quality/functionality of the response, which are increasingly recognized as important for protection and safety. Even though the reduced seropositivity in African Americans had suggested variations in vaccine efficacy across various ethnic groups, it was directly related to lower viremia, highlighting the fundamental role of the viral replication rate as an important driver of immunogenicity in that cohort. Rather than dismissing population-level differences, this finding underscores the need to better understand host and contextual factors when interpreting immunogenicity and extrapolating results across diverse populations. The high antibody response induced by TV003, particularly in orthoflavivirus-naive individuals, indicated its promise a vaccine candidate.

The TV005 formulation of the tetravalent vaccine, emerged as a significant advancement in the quest for an effective dengue vaccine, particularly because the efficacy of TV003 was limited by a lower DENV-2 replication rate and, consequently, seroconversion. TV005 is also a live attenuated tetravalent dengue vaccine designed to induce immunity against all four DENV serotypes, but with a higher potency of DENV-2 at 4.3 log10 PFU/ml compared to the TV003. Both formulations were compared in two follow-up placebo-controlled trials with orthoflavivirus-naive individuals^62^. Both vaccine candidates were well tolerated, with rash being the most common adverse event. TV005 vaccinees showed higher DENV-2 viremia, and while both candidates stimulated antibody production for all serotypes, TV003 had lower DENV-2 titers (76% compared to 97% for TV005)^62^.

### DV TV003/TV005

The outcomes of phase I monovalent studies were critical for the formulation and testing of the tetravalent TV003 vaccine candidate. A randomized study in subjects with documented prior exposure to orthoflavivirus demonstrated low viremia levels (0.68–1.1 log10 PFU/mL) and a tetravalent antibody response after one dose in 76% and 87% of the vaccinees, respectively^63^. Rash was the most common adverse event, along with headache, fatigue, and neutropenia, reinforcing the vaccine’s safety in orthoflavivirus-exposed individuals, as well as balanced and robust post-vaccination neutralizing antibody titers^63^.

A human challenge study on TV003-vaccinated subjects conducted six months post- vaccination, with the attenuated DENV-2 rDEN2Δ30 strain showed full protection (no viremia) at the 6-month challenge timepoint, supporting proof-of-concept protection in a controlled setting^64^. A similar TV005 study with attenuated DENV-2 rDEN2Δ30 and DENV-3 rDEN3Δ30 strains demonstrated similar outcomes^65^, reinforcing TV005’s safety and protection against infection. Importantly, because these challenges occurred approximately 6 months after vaccination, they support proof-of-concept protection in a controlled setting but do not demonstrate multi-year durability. These studies nonetheless highlight the value of CHIM for down-selecting candidates and informing the design of field efficacy/effectiveness evaluations.

Beginning in 2013, Butantan Institute partnered with the NIH for Phase II evaluations of TV003 admixture into the Butantan-DV program. Butantan-DV refers the Butantan-manufactured and lyophilized product derived from the NIH TV003 formulation^66^. Comparisons between ‘TV003’ with ‘Butantan-DV,’ were primarily to evaluate the impact of manufacturing/formulation and lot context (NIH-produced TV003 vs Butantan-produced Butantan-DV) on immunogenicity and safety, rather than to compare fundamentally distinct vaccine designs. TV005 was later developed as an optimized formulation of the same NIH platform to improve DENV-2 replication and enhance tetravalent balance relative to TV003. Despite this, the Butantan-DV program advanced with TV003 as the initial technology-transfer candidate for large-scale evaluation. Since manufacturing and formulation processes, including lyophilization, can affect vaccine-virus viability and immunogenicity, results from TV003 and Butantan-DV studies should be interpreted considering product and lot differences. Accordingly, the Phase II program incorporated an initial bridging step to compare Butantan-DV with TV003 in dengue-naïve participants^66^. From November 2013 to September 2015, 300 participants were enrolled in a safety and immunogenicity study where 1667 adverse events were reported within 21 days of immunization. Of these events, 833 were considered adverse reactions, mostly grades I and II, with no significant difference between vaccine or placebo groups. Common adverse reactions were headache, rash, and myalgia in those without prior dengue exposure, and rash, retroocular pain, and arthralgia in those with prior exposure. No severe adverse reactions were observed, and there was no significant difference between TV003 and Butantan-DV^66^.

The large sample size and diverse participant pool enhanced the robustness of the study outcomes. The high incidence of adverse events, particularly among previously exposed individuals, underscores the importance of detailed safety profiling. Overall, these studies support the tolerability and immunogenicity, and safety of TV003/TV005 and Butantan-DV across evaluated. The consistent safety, immunogenicity and efficacy across different formulations and populations reinforce the vaccine’s potential effectiveness in preventing dengue. But as with other dengue vaccine candidates, immunogenicity metrics (including seroconversion and neutralizing titers) should be interpreted as supportive but not sufficient to infer clinical protection without endpoint-driven efficacy/effectiveness data and functional immune profiling.

### Phase III study

For Phase III, efficacy was assessed for Butantan-DV (the Butantan-manufactured/lyophilized formulation derived from the NIH TV003 admixture; single-dose) in a double-blind trial conducted in Brazil including 16,235 participants from various locations across the country. Study participants were 2 to 59 years of age, of both sexes and previous exposure to any of the dengue serotypes. A single dose was administered, and participants were regularly monitored for adverse event and dengue occurrence. During follow-up, DENV-1 and DENV-2 were the predominant circulating serotypes, and only cases of DENV-1 and DENV-2 were identified in the study’s participants. The overall VE against symptomatic VCD (any serotype) was 79.6%, with serotype-specific VE was 89.5% against DENV-1, and 69.6% against DENV-2. When stratified by baseline serostatus, overall VE was higher, reaching 89.2% in previously exposed individuals and 73.6% in seronegatives. When stratified by age, the overall VE was 77.8% in adolescents (7–17 years), 80.1% in children (2–6 years), and 90% in adults (18–59 years). Notably, field efficacy against DENV-3 and DENV-4 could not be estimated within the observation window because these serotypes circulated at very low levels during the trial period, and therefore remain unmeasured in this dataset [47]. Longer-term follow-up is planned/ongoing to assess durability beyond two years and to virologically characterize breakthrough cases by serotype and genotype; analyses that will enable antigenic cartography to distinguish antigenic drift from simple waning immunity^46^.

As previously highlighted, a concern for any tetravalent dengue LAV is the possibility of unbalanced replication of its vaccine components, leading to immunodominance of one or two of its components and consequently, risk of breakthrough infections and severe dengue disease (SDD). Nivarthi et al. ^21^ elaborated further on the efficacy of dengue vaccines and proposed that while high neutralizing antibodies titers generally correlate with protection, their data showed poor correlation for serotype-specific neutralizing antibodies and efficacy in seronegative individuals. In a detailed immunoprofiling study of TV003 vaccine recipients, the study analyzed immune features associated with protection from DENV-2 in a controlled human infection setting. Using antigen-specific mapping, they reported that TV003 vaccination expanded DENV-2 reactive and cross-reactive antibody and memory B-cell repertoires and linked these features to reduced risk of post-challenge viremia. The study suggested that neutralizing antibodies targeting unique epitopes might be a better indicator of protective immunity than total neutralizing antibodies levels, as evidenced by the performance of the TV003. Serum analysis of 21 individuals who received a dose of the vaccine, showed robust replication of the vaccine components in most individuals, with varying levels of homotypic antibodies thus stimulating a neutralizing immune response. These observations confirm the immunologically balanced robustness of Butantan-DV corroborated previously^62,66,67^ and its but do not establish superiority over licensed vaccines without endpoint-driven comparative effectiveness data (Table 2); but importantly, however, immunological balance at early timepoints does not necessarily predict long-term, serotype-resolved field effectiveness. Although there have been no outbreaks to assess the efficacy for DENV-3, a study based on participants vaccinated with TV-005 and challenged by DENV-2 and DENV-3 six months later showed no viremia compared to placebo. These findings suggest the protective effect of the component for DENV-3^65^. Because this challenge was conducted at approximately 6 months, it supports short-interval proof-of-concept protection but did not establish durable protection against DENV-3 in the field, reinforcing the need for longer follow-up and serotype-resolved effectiveness data.

### Comparative Phase III Evidence: a Serotype-Resolved Matrix

To facilitate direct comparison among advanced tetravalent candidates, Table 3 presents platform/backbone, antigenic content (including NS proteins), dosing, regions and follow-up windows, prespecified endpoints, overall and serotype-resolved efficacy with 95% CIs, baseline serostatus and age effects, durability, safety signals, and assay conventions (PRNT_50/90_). In our assessment of public-health impact across licensed vaccines, we prioritize efficacy and effectiveness against clinically significant endpoints, specifically hospitalized dengue and severe dengue, and we explicitly indicate when values pertain to symptomatic VCD. The matrix also flags non-estimable serotypes (NA) when circulation was insufficient during follow-up and standardizes a “post-vaccination viremia ” readout tied to fixed post-dose timepoints. This “harmonized” view is intended to surface true between-platform differences that are obscured by mean neutralization titers alone and to support evidence-based design and policy decisions. Because surveillance intensity and case definitions can differ across trials, we highlight hospitalized VCD and SDD endpoints as the most clinically interpretable ‘like-for-like’ measures when comparing licensed vaccines and late-stage candidates.

### Other Candidates in Development

Beyond the three vaccines that have reached licensure or advanced phase III evaluation, several additional dengue vaccine strategies remain under active development. These approaches reflect a recognition that live-attenuated tetravalent constructs, while immunogenic, may not fully overcome the challenges of serotype imbalance, waning immunity, or safety concerns in seronegative populations. Alternative platforms are being pursued to diversify the dengue vaccine landscape, each with distinct immunological advantages and limitations. One promising avenue involves subunit vaccines, particularly those targeting the NS1. Unlike the E protein, NS1 is not incorporated into viral particles but is secreted abundantly during infection, where it contributes to immune evasion, complement activation, and vascular pathology^68^. Immunization with NS1 has demonstrated protection in animal models, reducing vascular leakage and mortality independent of neutralizing antibody responses^69^. These findings suggest that NS1-based vaccines could complement traditional approaches by targeting disease pathogenesis rather than viral entry. However, translating these results into durable human protection remains unproven, and concerns persist regarding potential autoimmunity triggered by NS1 cross-reactivity with host proteins^70^.

Nucleic acid platforms, particularly mRNA vaccines, have also entered the pipeline, inspired by the success of SARS-CoV-2 mRNA vaccines. Modified mRNA encoding the DENV E protein has conferred protection in animal models, inducing high titers of neutralizing antibodies and robust CD8⁺ T-cell responses^71^. These approaches offer advantages of rapid design, scalable production, and the possibility of fine-tuning antigenic expression. Early clinical evaluation is underway, although the extent to which mRNA vaccines can achieve balanced tetravalent immunity in humans remains unknown. DNA-based candidates encoding prM and E antigens have also shown immunogenicity in preclinical studies, though responses have been weaker than those achieved with live-attenuated or mRNA platforms^72^. Other strategies include viral-vectored vaccines, such as adenovirus or measles vectors encoding dengue antigens, designed to enhance cellular immunity^73^. While preclinical results are encouraging, progression to late-stage trials has been limited, in part due to competition from more advanced candidates and the logistical complexities of developing vector-based products for deployment in low-resource endemic regions. Collectively, these candidates highlight the evolution of dengue vaccinology from a singular focus on live-attenuated tetravalent constructs toward a more diversified portfolio. While none of these approaches have yet demonstrated the breadth of clinical evidence required for licensure, their development underscores the recognition that solving dengue’s immunological puzzle may require multi-pronged solutions that integrate humoral, cellular, and pathogenesis-modifying immune responses.

Vector targeted vaccine approaches have also been proposed as complementary strategies for arboviral diseases. Rather than targeting viral antigens directly, these approaches aim to elicit immune responses against mosquito salivary components that shape the early inoculation-site environment, with the goal of reducing saliva-mediated immunomodulation, altering local viral kinetics, and thereby lowering the probability of systemic infection and/or severe outcomes. Such strategies could, in principle, act as ‘pathogenesis-modifying’ or transmission-modifying adjuncts alongside conventional dengue vaccines, particularly because most vaccine evaluations rely on needle inoculation and may not capture saliva-dependent immune effects. However, vector-targeted vaccines remain comparatively early in development, and key uncertainties include antigen selection, durability, breadth across mosquito populations/species, and how anti-saliva immunity would integrate with dengue-specific vaccine responses^74,75^.

## Cross-cutting Immunological Lessons from Dengue Vaccines

The collective experience with Dengvaxia®, Qdenga®, and Butantan-DV has illuminated fundamental immunological principles that continue to shape the trajectory of dengue vaccinology. Perhaps the most critical lesson is that neutralizing antibody titers, while indispensable for protection, are not sufficient in isolation to predict clinical efficacy. Multiple clinical trials and cohort studies have demonstrated that individuals with high titers can nevertheless experience breakthrough infection, particularly when exposed to serotypes other than those most robustly targeted by the vaccine^35,36,76,77^. This discrepancy underscores that protection against dengue depends on the quality of the immune response, conceptualized as a multidimensional construct encompassing (i) epitope specificity, including quaternary-epitope targeting, (ii) binding strength and maturation, such as avidity, (iii) Fc-mediated effector functionality, and (iv) durability and recall features^78^. Equally important is the recognition that balanced serotype coverage is the central design constraint for any dengue vaccine. The contrasting outcomes of different platforms illustrate this point: CYD-TDV elicited responses dominated by DENV-4, while TAK-003 induced robust and durable immunity primarily against DENV-2, resulting in heterogeneous field efficacy^27,28,35^. In contrast, Butantan-DV TV003/TV005 achieved a more balanced early replication and tetravalent neutralizing antibody profile. However, balanced immunogenicity does not guarantee uniformly durable field protection across serotypes and baseline serostatus. Extended follow-up revealed a decline in overall vaccine efficacy compared to the 2-year interim analysis, with efficacy estimates driven by DENV-1 and DENV-2 circulation, and DENV-3/DENV-4 efficacy remaining unestimated due to insufficient circulation^18,21,46,79^. Achieving this equilibrium is complex, as interference among live-attenuated backbones can favor replication of certain strains, yet it is essential for consistent protection in hyperendemic regions where all serotypes co-circulate. In this context, ‘balanced immunity’ refers to the absence of marked serotype dominance in protective immune features, such as neutralization potency and functional antibody profiles, that could leave one or more serotypes under-protected. Notably, the field lacks a standardized definition and prespecified thresholds for ‘balance,’ complicating cross-trial comparisons and interpretation of serotype-specific performance.

Another lesson repeatedly emphasized is the profound impact of baseline serostatus. CYD-TDV was effective in boosting immunity among previously exposed individuals but paradoxically increased the risk of hospitalization and severe dengue in baseline seronegatives, in long-term follow up^12,30^. Multiple mechanisms have been proposed to explain this observation (including ADE), but direct mechanistic confirmation in vaccine recipients remains challenging. These findings prompted major changes in recommendation toward serostatus-informed use. This sobering outcome clarified that vaccination in dengue-naive populations carries unique risks if the induced antibodies are cross-reactive yet sub-neutralizing, a reality that led WHO and other stakeholders to revise dengue vaccine policy toward pre-vaccination serostatus screening and more restrictive risk-benefit implementation strategies^31^. By contrast, both TAK-003 and Butantan-DV have thus far avoided this pitfall, performing safely in seronegative cohorts, although continued long-term follow-up is essential to confirm these trends.

Beyond the magnitude of antibody responses, their quality has emerged as equally decisive. Studies of human monoclonal antibodies and vaccine sera have shown that antibodies targeting quaternary epitopes displayed on assembled E-protein dimers, such as EDE-like specificities, confer broader and more potent protection than those directed at monomeric domains or conserved fusion-loop regions, which are often weakly neutralizing or enhancing^80–82^. Vaccine platforms that can consistently drive such quaternary-epitope responses (through stabilized antigens, engineered dimers, or optimized replication balance) are therefore more likely to generate the breadth and safety profile required for universal deployment. Cellular immunity also plays a non-negligible role in shaping outcomes. Natural infection induces vigorous CD8⁺ T-cell responses targeting conserved nonstructural proteins, such as NS3 and NS5, responses largely absent in CYD-TDV because of its reliance on the YF17D backbone^28,29^. TAK-003 and Butantan-DV, which retain dengue NS antigens, elicit more substantial dengue-specific cellular immunity than CYD-TDV. For TAK-003, NS-directed responses are expected to be enriched toward the DENV-2 backbone, but available data support cross-reactive CD4⁺/CD8⁺ responses to conserved NS epitopes across serotypes; Butantan-DV, by design, includes dengue NS proteins in each component^18,83,84^. Although defining the precise contribution of T cells to clinical protection remains challenging, accumulating evidence suggests that durable, polyfunctional cellular responses complement humoral immunity and may extend protection as neutralizing antibody titers wane.

An additional insight relates to post-vaccination viremia and the replication dynamics of live-attenuated formulations. Balanced, moderate replication of each component appears critical to eliciting trivalent or tetravalent responses. A trivalent profile may still confer meaningful protection in specific settings or time windows; however, missing coverage for any serotype can leave a residual risk that becomes important when serotype circulation shifts or multiple serotypes co-circulate^27,28,36^. In this regard, the performance of Butantan-DV in both field trials and controlled human infection models, where vaccinees exhibited sterilizing protection against DENV-2 and DENV-3 challenge, provides proof of concept that replication balance can support short-term protection in controlled human infection models. However, translating CHIM findings into durable field effectiveness particularly across serotypes, genotypes, and multi-year follow-up requires caution, as durability, antigenic mismatch, and formulation or population differences may contribute to reduced long-term effectiveness despite early sterilizing protection^18,21,46,64,65,79^. These observations converge on the need for composite correlates of protection that integrate humoral and cellular metrics. Reliance on plaque-reduction neutralization tests alone has proven insufficient, as they fail to capture the quality, durability, and functional breadth of responses. More reliable correlates will likely combine neutralization titers with assessments of antibody avidity, memory B-cell repertoires, and T-cell functionality, ideally validated against both real-world clinical endpoints and data from controlled human infection models^64,65,76,81^. Establishing such correlates is not merely academic; it is essential for guiding next-generation vaccine design and for interpreting efficacy across diverse epidemiological contexts.

Finally, the role of the vector cannot be ignored. A growing body of evidence shows that mosquito saliva profoundly modulates early immune responses, enhancing local viral replication, altering cytokine profiles, and reshaping adaptive immunity for days after the bite^74^. Methodologically, most vaccine studies use needle inoculation, which may oversimplify the complexity of natural transmission. Incorporating vector-aware designs into preclinical models and accounting for saliva-derived immunomodulation in vaccine evaluation are necessary to fully understand efficacy and optimize future interventions. Collectively, these lessons indicate that dengue vaccines must move beyond the pursuit of high neutralizing titers alone and adopt a more integrated immunological approach. Effective candidates will need to achieve balanced serotype coverage, induce antibodies targeting protective quaternary epitopes, elicit durable T-cell responses, and demonstrate safety across serostatus groups while considering the biology of natural transmission. These principles, derived from extensive experience, offer a roadmap for next-generation dengue vaccines and serve as a template for vaccinology against other complex arboviruses. This discussion does not suggest that pre-existing anti-saliva immunity necessarily alters vaccine-induced responses or advocate for the inclusion of salivary antigens in dengue vaccines; rather, it highlights an evaluation gap that could be addressed by vector-inclusive preclinical models and refinements to CHIM.

## Unresolved Questions and Correlates of Protection

Despite significant advances, several fundamental questions in dengue vaccinology remain unresolved, and addressing these issues is essential for the rational design of next-generation vaccine candidates. Foremost among these challenges is the definition of reliable correlates of protection. In this context, a ‘correlate of protection’ (CoP) refers to an immune marker that is statistically associated with a reduced risk of a prespecified clinical endpoint. Correlates may be non-mechanistic (predictive but not necessarily causal) or mechanistic (causally involved in protection), and require external validation across diverse populations, serostatus groups, and time periods. For decades, PRNTs have been considered the gold standard for evaluating dengue vaccine immunogenicity, based on the assumption that higher titers of serotype-specific neutralizing antibodies directly confer protection. However, data from all three major vaccines indicate that this correlation is inconsistent. For example, Dengvaxia® induced high neutralization titers but failed to protect seronegative individuals, and TAK-003 generated robust titers to DENV-2 without achieving durable cross-serotype efficacy^24,30,35,36^. Even the Butantan-DV, which demonstrated more balanced replication and immunogenicity, revealed that neutralizing antibodies alone do not fully account for protection. This is particularly evident given that serotype-specific VE for DENV-3 and DENV-4 could not be estimated when circulation was insufficient, and extended follow-up has shown declining overall VE over time^46^. These findings indicate that not all neutralizing antibodies have equivalent functional consequences. Antibodies targeting quaternary epitopes on virion-assembled E dimers can provide potent and broadly protective immunity, whereas those directed against monomeric domains or the conserved fusion loop may be weakly neutralizing or even enhancing^80^. Current assays do not discriminate between these antibody populations, leading to overestimation of protection when titers are dominated by cross-reactive, low-avidity specificities. Refinement of serological assays to incorporate measures of antibody quality, avidity, and epitope specificity is therefore critical for identifying protective signatures that extend beyond simple quantitative neutralization. To address the limitations of PRNT-only readouts, Table 4 presents a model for a minimal composite panel intended to clarify prospective testing, although it is not yet a validated or regulatory-accepted correlate. While systems serology has identified Fc-feature and effector-function signatures associated with protection in natural infection cohorts, similar composite panels have not yet been consistently validated against clinical endpoints in dengue vaccine trials. This underscores the need for harmonized, prospective evaluation. The table details operational metrics, feasible multicenter assays, sampling windows, and example decision rules, thereby enabling prospective validation and immunobridging analyses while ensuring that each component remains explicitly falsifiable and trial-agnostic.

Another unresolved dimension concerns the role of cellular immunity. Multiple cohort studies and experimental models demonstrate that CD8⁺ T-cell responses, particularly against nonstructural proteins, such as NS3 and NS5, contribute to viral clearance and may provide cross-serotype protection^18,29^. These responses are not captured by PRNTs and are often under-characterized in clinical trials. The absence of NS antigens in Dengvaxia® provides a striking natural experiment: its limited induction of CD8⁺ T cells plausibly contributed to serostatus-dependent efficacy, contrasting with TAK-003 and Butantan-DV, which include dengue NS proteins and appear to elicit more robust cellular responses^28,83^. However, the precise magnitude and functional quality of these T-cell responses required for durable protection remain undefined, complicating efforts to establish composite correlates. The durability of vaccine-induced immunity also remains a pressing concern. Long-term follow-up of TAK-003 revealed declining efficacy over 3–4.5 years, with disproportionate loss of protection against non-DENV-2 serotypes^35,36^. Whether waning of protection reflects contraction of antibody titers, narrowing of memory B-cell repertoires, inadequate T-cell help, or a combination thereof is not yet clear. In the absence of mechanistic correlates, durability cannot be reliably predicted, leaving programmatic uncertainty for vaccine deployment in endemic settings where multiserotype exposure is inevitable.

Adding further complexity, the context of natural infection transmitted through the bite of *Aedes* mosquitoes has been largely absent from vaccine evaluation. Experimental work shows that mosquito saliva profoundly modulates early host responses, enhancing local viral replication, altering cytokine landscapes, and reshaping the trajectory of adaptive immunity^22,75,85^. Because virtually all clinical studies to date have used needle inoculation, the extent to which salivary factors may alter vaccine performance remains unknown. Incorporating vector-derived modulation into preclinical models and controlled human infection studies will be essential to ensure that correlates of protection reflect the realities of natural transmission. Finally, the phenomenon of orthoflavivirus cross-reactivity continues to confound vaccine evaluation. In endemic regions, individuals are often sequentially exposed to multiple orthoflaviviruses, including Zika and yellow fever, which can redirect B- and T-cell repertoires toward conserved epitopes shared with dengue^20,86^. Such cross-reactivity may bias immune responses in ways that are difficult to predict, sometimes broadening immunity, but at other times narrowing specificity or promoting ADE. Until correlates of protection are defined that explicitly account for these immunological interactions, the performance of dengue vaccines will remain context-dependent and difficult to generalize globally.

## Perspective and Future Directions

The trajectory of dengue vaccine development offers both a roadmap and a cautionary tale for future arbovirus vaccinology. After more than half a century of effort, three advanced candidates (CYD-TDV, TAK-003, and Butantan-DV) have demonstrated that effective vaccination against dengue is possible, yet each has also underscored the formidable immunological constraints that must be overcome. The lessons distilled from these experiences extend beyond dengue itself, informing strategies for vaccine development against other complex vector-borne viruses where immunity is multifaceted and enhancement remains a risk. Looking forward, the central imperative is to define correlates of protection that integrate both humoral and cellular immunity. Neutralizing antibodies will remain indispensable, but they must be contextualized with measures of epitope specificity, avidity, and the durability of memory B-cell pools^76,77,80^. In parallel, CD8⁺ and CD4⁺ T-cell responses, particularly those directed against conserved nonstructural proteins, must be incorporated into standardized correlates, as they contribute to viral clearance and likely provide resilience as antibody titers wane^18,29^. Only through such composite markers, validated in longitudinal cohorts and controlled human infection models, can vaccine evaluation move from empirical observation to predictive immunology. The durability of protection represents another pressing frontier. Even promising candidates, such as TAK-003 have demonstrated declining efficacy within 3–4.5 years, disproportionately affecting non-dominant serotypes^36^. Strategies to extend durability may include heterologous prime–boost regimens, rational antigen engineering to stabilize quaternary epitopes, and adjuvant formulations that enhance germinal center responses and long-lived plasma cell induction^81,82^. Advances in systems immunology and high-dimensional profiling can guide these efforts by identifying immune signatures predictive of persistence, thereby informing rational design and booster strategies.

The role of natural transmission by mosquitoes cannot be overlooked. A growing body of evidence shows that mosquito saliva shapes the immunological microenvironment of infection, modulating innate responses, altering cytokine landscapes, and facilitating viral dissemination^22,75^. Future preclinical models and even human challenge studies should incorporate vector-derived components or saliva-mimetic adjuvants to better approximate natural infection. By ignoring this dimension, current vaccine evaluations may underestimate the complexity of immune priming in endemic settings. Another frontier lies in next-generation platforms. Live-attenuated vaccines will remain central given their ability to mimic natural infection and induce broad immunity, but alternative strategies including mRNA vaccines, viral vectors, and NS1-based subunit approaches offer opportunities to diversify the immune response and reduce risks associated with ADE. Hybrid platforms that combine structural and nonstructural antigens, or leverage sequential heterologous immunizations, may ultimately provide the most balanced and durable protection. Lessons from the rapid development of SARS-CoV-2 vaccines also highlight the value of flexible, scalable platforms that can be updated as needed in response to genotype replacement and antigenic variation within DENV serotypes, supported by ongoing genomic and antigenic surveillance^87,88^. Vaccine implementation must be tailored to epidemiological realities. Baseline serostatus will continue to influence benefit–risk calculations, especially in regions with variable seroprevalence. Diagnostic tools capable of rapid and accurate serostatus determination will therefore be critical for guiding deployment of existing vaccines, such as Dengvaxia®. At the same time, the goal should be to advance candidates that are safe and effective regardless of serostatus, thereby simplifying programmatic use and enabling equitable access in resource-limited settings.

The road ahead therefore requires a deliberate shift: from empiricism to predictive immunology, from reliance on singular endpoints to composite correlates, and from conventional platforms to flexible designs capable of sustaining balanced and durable immunity. Advances in structural vaccinology, mRNA technology, and systems immunology provide the tools to meet these demands, but their application must be guided by the lessons, sometimes costly, that dengue has already taught. Dengue vaccinology now serves as both a case study and a paradigm, illustrating that the development of safe and effective vaccines for complex arboviruses requires a comprehensive understanding of immunological complexity. At the same time, vaccine development must address financial, manufacturing, and logistical limitations, which demand pragmatic study designs and scalable technological solutions.

### Ethics approval and consent to participate

Not applicable. This article is a narrative review and did not involve any new studies with human participants or animals performed by any of the authors. Where human studies are discussed, they were conducted in accordance with internationally accepted standards (e.g., the Declaration of Helsinki) and the original publications report approval by an appropriate ethics committee.

## Data Availability

No datasets were generated or analyzed during the current study.
